# Copper-mediated MAM regulation of the NF-κB signalling pathway enhances Seneca Valley virus replication in PK-15 cells

**DOI:** 10.1186/s13567-025-01578-w

**Published:** 2025-09-19

**Authors:** Xiaoli Wu, Song Zhu, Min Li, Zhiwen Xu, Ling Zhu, Junliang Deng, Huidan Deng

**Affiliations:** 1 College of Veterinary Medicine, Sichuan Agricultural University, Wenjiang, Chengdu, 611130 China; 2 Key Laboratory of Animal Diseases and Environmental Hazards of Sichuan Province, College of Veterinary Medicine, Sichuan Agricultural University, Wenjiang, Chengdu, 611130 China

**Keywords:** SVV, copper homeostasis, MAM, NF-κB pathway, innate immunity

## Abstract

Seneca Valley virus (SVV) is known to cause vesicular disease in swine, presenting new challenges to the pig industry. Recent studies have investigated the relationship between disrupted copper ion homeostasis and viral replication, suggesting that copper dysregulation has a significant impact on the replication of various viruses. Research has also shown that mitochondria-associated endoplasmic reticulum membrane (MAM) and NF-κB are involved in the innate immune response triggered by viral infections. However, the exact mechanisms by which copper (Cu), MAM, and NF-κB affect SVV replication remain unclear. In this study, it was found that SVV induces an imbalance in copper homeostasis, leading to dynamic changes in MAM while inhibiting the NF-κB pathway. This inhibition results in decreased levels of IL-6, IL-1β, TNF-α, IFN-α, and IFNλ3. Furthermore, the disruption of copper homeostasis in SVV-infected PK-15 cells regulates the NF-κB pathway through MAM, promoting SVV replication. This research provides valuable insights into the regulation of copper metabolism during SVV infection and establishes a theoretical framework for understanding the pathogenesis and immune activation mechanisms associated with SVV.

## Introduction

Seneca Valley virus (SVV) is the only known member of the *Senecavirus* genus within the *Picornaviridae* family. It became a significant concern in 2007 after an outbreak of swine vesicular disease in Canada [1]. The first case of SVV infection in China was identified in Guangdong Province in 2015, characterised by vesicular lesions in sows and the acute death of neonatal piglets [2]. Following this initial outbreak, infections were subsequently reported on six different farms in Guangdong and Hubei Provinces from 2015 to 2016, as well as in other regions [3]. This virus has caused substantial economic losses to the global pork industry [4]. To date, there are no medications or vaccinations currently available to prevent SVV. Therefore, research into the disease's aetiology is critical for better identification and the establishment of effective prevention and control strategies.

Copper (Cu) is a vital trace element that plays a crucial role in numerous physiological processes. It is transported into cells from the extracellular space by the CTR1 protein through the Atox1-ATP7A/B-Cp pathway. Once inside, copper is pumped into the bloodstream and used in the ER, with a significant portion stored in the mitochondria [5, 6].

Disruptions in copper homeostasis are pivotal in the development of several diseases, including Menkes disease and Wilson disease [7], Alzheimer’s disease [8], certain types of cancers [9], and connective tissue disorders [10]. Additionally, research has indicated that copper may play a crucial role in fighting viral diseases.

For instance, Cu inhibits the replication of the dengue virus in Vero cells [11]. In contrast, in HIV-infected CD4 T cells, an accumulation of copper is associated with increased viral replication [12]. Cu chelators have been shown to reduce the replication of influenza A in lung cells, and depleting CTR1 significantly decreases viral RNA synthesis [13]. Moreover, after Zika virus infection, Cu chelators promote cell survival without lowering the percentage of infected cells or viral load [14].

In conclusion, copper may possess antiviral properties, facilitate viral replication, or have no apparent effect on viral proliferation. However, the precise role of copper in the replication of the SVV virus remains to be fully understood.

Innate immunity serves as the first line of defence against invading pathogens. During a viral infection, host pattern recognition receptors (PRRs) detect pathogen ligands and recruit pertinent receptor molecules, triggering the production of interferons (IFNs) and inflammatory factors. This process is mediated by interferon regulatory factors (IRFs) and nuclear factor κB (NF-κB) [15]. Under normal physiological conditions, NF-κB resides in the cytoplasm in an inactive state, bound to the inhibitory protein IκB. When stimulated, IκB is ubiquitinated and degraded by the proteasome, leading to a decrease in IκB levels. This degradation releases NF-κB, enhancing its transcriptional activity and the activity of genes encoding IFNs and other pro-inflammatory factors [16].

Numerous studies indicate that SVV disrupts host defence mechanisms within virus-infected cells. In human embryonic kidney 293 T cells, SVV inhibits the production of host IFNs by targeting MAVS, TRIF, and TANK to facilitate TRAF6-induced NF-κB activation [17]. Additionally, a highly dynamic communication system exists between the endoplasmic reticulum (ER) and mitochondria, known as the mitochondria-associated endoplasmic reticulum membrane (MAM). This membrane contains several key receptor molecules, including MAVS [18].

Accumulating evidence suggests that MAM not only regulates physiological functions such as inflammatory responses, cellular autophagy, and phospholipid synthesis, but also plays a crucial role in viral pathogenesis and antiviral immune responses [19].

Mfn2, a resident protein of MAM, maintains its structure and function while regulating innate immune responses during viral infections [20–22]. Research has shown that overexpressing Mfn2 can suppress dengue virus replication by modulating mitochondrial function and cellular apoptosis [11, 23]. Conversely, reducing Mfn2 expression decreases IL-1β secretion in virus-infected macrophages, subsequently inhibiting viral replication [24].

Our previous study also confirmed that in PK-15 cells infected with SVV, Mfn2 protein expression was inhibited [25]. However, the precise mechanism by which SVV affects MAM and the relationship between MAM and NF-κB remain unclear.

We hypothesise that copper homeostasis may play a role in the replication of SVV. Furthermore, copper could influence SVV replication by modulating the MAM and NF-κB pathways. Our findings suggest a novel mechanism of SVV infection, offering new insights for the prevention and treatment of SVV.

## Materials and methods

### Materials and reagents

CuSO_4_ was purchased from Chengdu Kelong Chemical Co., Ltd. (Chengdu, China). The chemical reagents, ammonium tetra-thiomolybdate, and TTM were purchased from Sigma Aldrich (15,060–55-6). Mitochondrial fusion promoter M1(S3375) and JSH-23(S7351) were obtained from Selleck (Shanghai, China). The ELISA kits were purchased from Jiangsu Jingmei Biotech, including the IL-1β ELISA Research Kit 96 T (JM-01256P1), IL-6 ELISA Research Kit 96 T (JM-01252P1), IFN-α ELISA Research Kit 96 T (JM-01212P1), IFN-λ3 ELISA Research Kit 96 T (JM-10518P1), and the TNF-α ELISA Research Kit 96 T (JM-01217P1).

Rabbit MFN2 polyAb(12,186–1-AP), rabbit SLC31A1 polyAb (27,499–1-AP), rabbit ATOX1 polyAb (22,641–1-AP), rabbit COX17 polyAb (11,464–1-AP) and rabbit ATP7A (abs1499536) were obtained from Proteintech (Wuhan, China). Rabbit NF-κB p65/Rel A mAb (A22331), rabbit phospho-NF-κB p65/RelA-S536 mAb (AP1294), rabbit IκBα mAb (A24909) and HRP-conjugated Goat anti-rabbit IgG (H + L) were purchased from Abclonal (Wuhan, China). anti-β-actin (66,009–1-Ig, Proteintech, American).

### Cell culture and treatments

PK-15 cells, kept at the Key Laboratory of Animal Disease and Human Health of Sichuan Province at Sichuan Agricultural University in Chengdu, were maintained in Dulbecco’s Modified Eagle’s Medium Nutrient Mixture (DMEM) (Gibco, USA). The medium was supplemented with antibiotics (100 units/mL penicillin and 100 μg/mL streptomycin) and 10% Newborn Bovine Serum (NBS) (abs978, Absin, Shanghai, China). The SVV was also maintained at the Key Laboratory of Animal Disease and Human Health of Sichuan Province. The virus was propagated in PK15 cells using DMEM supplemented with 2% NBS.

### Assessment of cell viability

The viability of PK-15 cells was assessed using the CCK-8 assay. PK-15 cells were seeded into 96-well plates and treated separately with SVV, various concentrations of TTM, CuSO_4_, the mitochondrial fusion promoter M1, and JSH-23 for 24 h. After treatment, the cells were incubated with CCK-8 (ST316, Beyotime, Shanghai, China) at 37 °C for 1.5 h. The optical density (OD) was measured at 450 nm using a microplate reader to reflect cell viability. The ideal dose was determined by evaluating the inhibitory concentrations of TTM, CuSO_4_, the Mitochondrial Fusion Promoter M1, and JSH-23 on PK-15 cells.

### Western blot

To extract protein from PK-15 cells, the procedure outlined in the Cell Isolation Kit (Beyotime) was followed. After adding lysate (PMSF was added before clinical use) to PK-15 cells, the mixture was homogenised and centrifuged to collect the supernatant. Next, a sample buffer was added to isolate the total protein from the PK-15 cells.

Protein quantification was performed according to the manufacturer's instructions using the BCA Protein Assay Kit (Beyotime, Shanghai, China). After preparing the gel and samples, performing electrophoresis, transferring the proteins to membrane, performing the immunoreaction, and conducting the colour reaction, we obtained the protein band. Finally, we used ImageJ software to analyse significant differences in protein expression.

### Immunofluorescence assay

To prepare the working solution, ER-Tracker Green and MitoSOX™ Red were diluted with ER Tracker Green diluent and DMEM, respectively, at ratios of 1:1000 and 1:10 000. The cells were then incubated with 60 μL and 100 μL of the working solution at 37 °C in the dark for 25 min and 30 min, respectively. After the incubation period, the cells were washed three times with PBS and fixed with 4% paraformaldehyde for 5 min at room temperature (RT). Finally, the cells were mounted using an anti-fluorescence quenching mounting medium containing DAPI and visualised directly under a fluorescence microscope.

### Quantitative real-time PCR (RT-qPCR)

RNA was extracted from PK-15 cell pellets using an RNA extraction kit from FOREGENE (Chengdu, China). Following extraction, the RNA samples were reverse-transcribed into complementary DNA (cDNA) using the Reverse Transcriptase Kit purchased from FOREGENE (Chengdu, China), adhering strictly to the manufacturer’s guidelines. For gene expression analysis, quantitative PCR (qPCR) was conducted utilising Genius 2X SYBR Green Fast qPCR Mix, obtained from Abclonal (Wuhan, China). The copy number of SVV was determined using the standard curve equation: y = -3.6020x + 39.18. At least three biological replicates were analysed for each experiment. The RT-qPCR primer is shown in Table 1.

**Table 1:** **Primer sequences of SVV.**

Primer name	Forward primers (5'-3')	Reverse primers (5'-3')
SVV VP1	AGGTACTGGAGAAGGACGCT	GGTTGACGTACAGGCCGAAA

### Enzyme-linked immunosorbent assay (ELISA)

PK-15 cells were inoculated into 96-well plates and subsequently treated with SVV, TTM, CuSO_4_, the Mitochondrial Fusion Promoter M1, and JSH-23 for 24 h. After the treatment, supernatants were collected and centrifuged at 3000 rpm for 10 min for ELISA. The levels of IL-6, IL-1β, TNF-α, IFN-α, and IFNλ3 in the supernatants were measured using ELISA kits according to the manufacturers’ protocols. Optical densities were read at 450 nm. All experiments were performed in triplicate, and the results for IL-6, IL-1β, TNF-α, IFN-α, and IFNλ3 were expressed as concentrations in pg/mL.

### Copper ion concentration detection

PK-15 cells were inoculated into 6-well plates and subsequently treated with SVV, TTM, and CuSO_4_. To determine the concentration of Cu within the medium of PK-15 cells, the Cell Copper Colorimetric Assay Kit was used. The OD value was then measured, and a calculation formula was used to calculate the copper ion content.

### Statistical analysis

Data are presented as mean ± standard deviation. All statistical analyses were conducted using GraphPad Prism (GraphPad Software, Inc.). Unpaired T-tests and one-way analysis of variance (ANOVA) were employed to assess the significance of differences between the experimental groups and the control group. *P* < 0.05 indicates statistical significance. Each experiment was repeated at least three times.

## Results

### SVV causes imbalance in copper ion homeostasis

**Figure 1:**
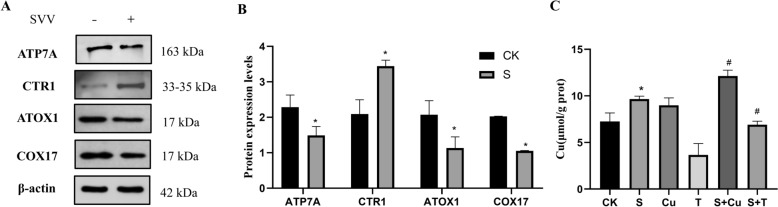
**Expression of copper transport system-related proteins and copper concentration in PK-15 cells infected by SVV**. **A Western blot assay of ATP7A, CTR1, ATOX1 and COX17**. **B Relative protein expression levels of ATP7A, CTR1, ATOX1 and COX17**. **C Intracellular copper ion concentration by Cell Copper Colorimetric Assay Kit. “CK” means control group, “T” means TTM group and “Cu” means CuSO**_**4**_ group. “S + T” means the SVV group with TTM, and “S + Cu” means the SVV group with CuSO4. Data are presented with the means ± standard deviation. * P **< 0.05 compared with the control group. #* P*** **< 0.05, compared with the SVV group**.

The results of the Western blot analysis, as shown in Figures 1A and B, indicate a significant decrease in the expression levels of ATP7A, ATOX1 and COX17 in SVV-infected PK-15 cells compared to the control group (*P* < 0.05). In contrast, the protein abundance of CTR1 increased notably (*P* < 0.05). Additionally, the Cell Copper Complication assay was conducted to measure the intracellular copper content, revealing that SVV infection leads to a substantial increase (*P* < 0.05) in Cu^2+^ concentrations (Figure 1C). The introduction of an additional 100 μM CuSO_4_ further elevated intracellular copper levels. However, administration of 1 mM TTM significantly reduced (*P* < 0.05) these levels compared to the SVV group. These findings suggest that SVV promotes the accumulation of copper ions in PK-15 cells.

### SVV causes MAM dysfunction in PK-15 cells

**Figure 2:**
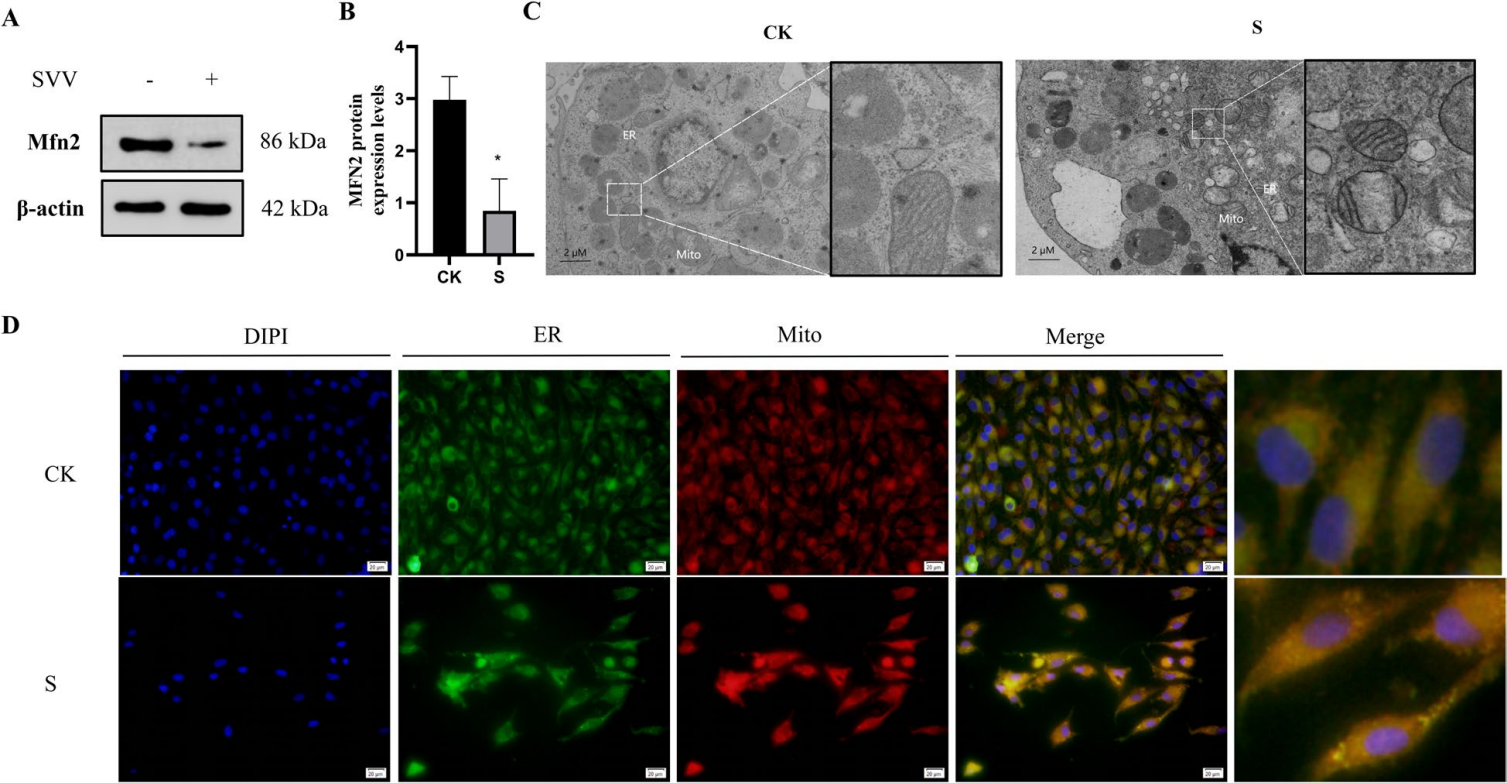
**SVV-induced MAM dysfunction in PK-15 cells. A Western blot assay of Mfn2.** **B Relative protein expression levels of Mfn2.** **C Ultrastructure of the PK-15 cells, the scale bar is 2 or 1 μM. “Mito” means mitochondrion, and “ER” means endoplasmic reticulum.** D The observation of MAMs by Immunofluorescence. Data are presented with the means ± standard deviation. * P **< 0.05 compared with the control group.**

The abundance of the Mfn2 protein, a crucial regulatory protein of MAM, was evaluated using Western blot analysis (Figures 2A and B). This analysis revealed a significant reduction (*P* < 0.05) in the SVV group relative to the control group.

To further investigate the morphological changes of MAM in PK-15 cells, we conducted immunofluorescence assays and transmission electron microscopy (TEM). The TEM results showed that mitochondria in the SVV group displayed signs of vacuolation and spine fractures in contrast to the control group. Furthermore, the distance between the ER membrane and the mitochondrial outer membrane was reduced (Figure 2C).

Additionally, after incubating the cells with Mito-Tracker Red and ER-Tracker Green, we observed changes in MAM using a fluorescence microscope (FM) (Figure 2D). The results indicated that the fluorescence signal at the junction where the ER and mitochondria interact was enhanced compared to the control group. These findings suggest that SVV disrupts the structure of MAM.

### Copper ion homeostasis imbalance affects SVV virus replication by regulating MAM in PK-15 cells

**Figure 3:**
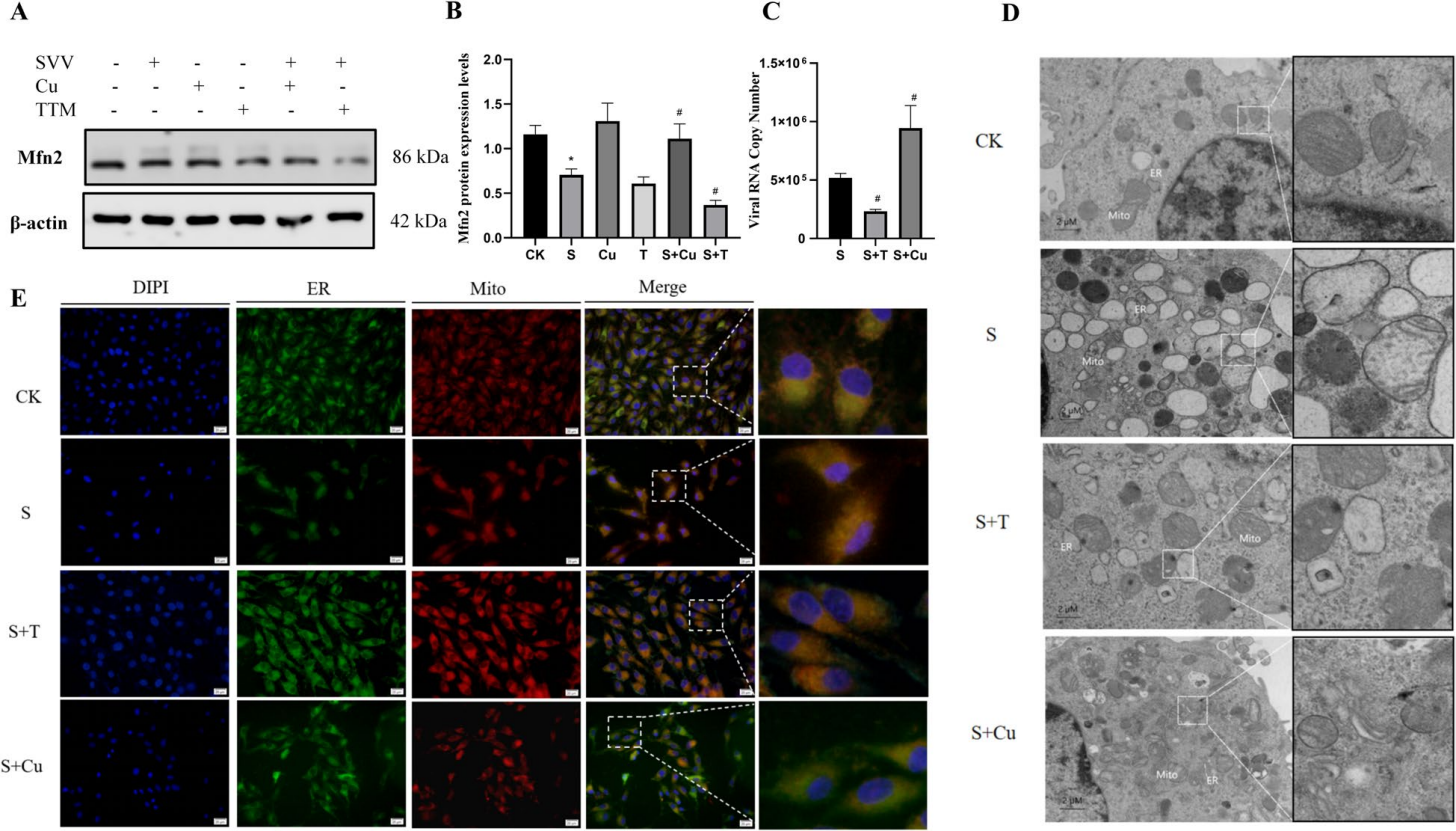
**Copper ion homeostasis imbalance affects SVV virus replication by regulating MAM in PK-15 cells**. A-B Protein expression levels of Mfn2 in PK-15 cells. C The viral copy number of SVV in PK-15 cells. D Ultrastructure of the PK-15 cells. E Immunoblot analysis of MAM in PK-15 cells. Data are presented with the means ± standard deviation. * P **< 0.05 compared with the control group. #* P*** **< 0.05 compared with the SVV group.**

Western blot analysis results (Figures 3A and B) indicated that the administration of 100 μM CuSO_4_ significantly increased (*P* < 0.05) Mfn2 protein compared to the SVV group. In contrast, treatment with 1 mM TTM resulted in a marked decrease (*P* < 0.05) in Mfn2 expression in SVV-infected PK-15 cells. Additionally, while the viral copy number in the SVV-infected PK-15 cell group decreased following treatment with TTM, there was a significant increase (*P* < 0.05) in the viral copy number when these cells were treated with copper (Figure 3C).

TEM results (Figure 3D) confirmed that the addition of TTM to SVV-infected PK-15 cells reduced the intermembrane distance compared to the SVV group. However, severe mitochondrial vacuolation was observed in the SVV and copper co-treatment group, where the distance between the ER and the mitochondrial outer membrane was greater than in the SVV group. Additionally, we employed fluorescence colocalization to examine SVV-infected PK-15 cells exposed to TTM or Cu^2+^ (Figure 3E). With the addition of TTM, the interaction area expanded; in contrast, the fluorescence signal intensity decreased significantly after adding copper compared to the SVV group. These findings shed light on the potential mechanisms through which copper may upregulate Mfn2 expression, thereby exacerbating MAM dysfunction caused by SVV and promoting SVV replication.

### Copper ion homeostasis imbalance affects SVV virus replication by regulating NF-κB signalling in PK-15 cells

**Figure 4:**
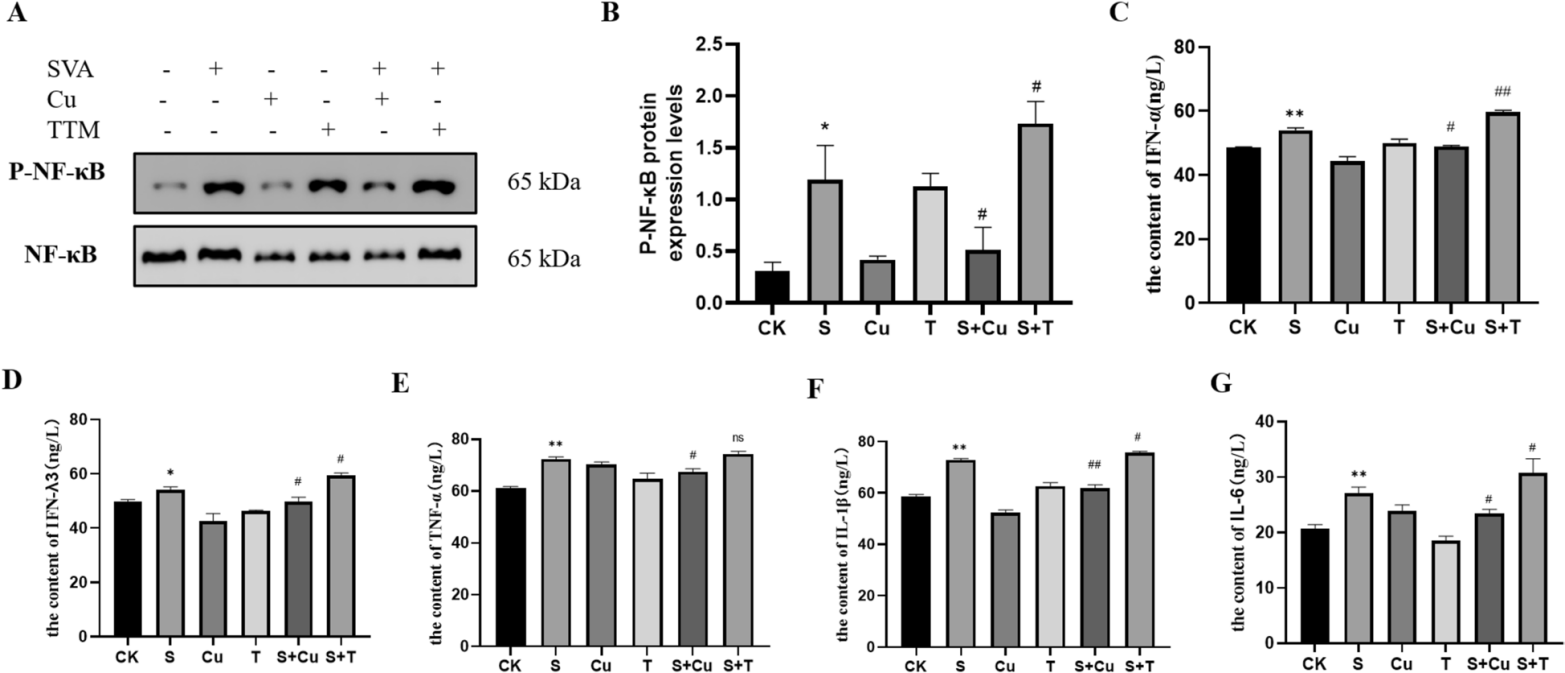
**N. A–B Protein expression levels of p-NF-κB in PK-15 cells. C-G The content of IL-1β, IL-6, TNF-α, IFN-α, and IFN-λ3 (F-κB signalling pathway in PK-15 cells infected with SVV*n*** = 3). Data are presented with the means ± standard deviation. * P < 0.05, ** P **< 0.01 compared with the control group. #* P*** **< 0.05, ##* P*** **< 0.01 compared with the SVV group.**

Viruses can activate the NF-κB signalling pathway, leading to the production of inflammatory mediators and interferons (IFNs) in response to viral infections. Our results, as shown in Figures 4A and B, indicated that the expression of phosphorylated NF-κB (p-NF-κB) protein was significantly elevated (*P* < 0.05) in the SVV group compared to the control group. When we supplemented the SVV group with Cu and TTM individually, we observed a notable decline (*P* < 0.05) in p-NF-κB levels with Cu, while TTM caused a significant increase (*P* < 0.05).

Additionally, we used ELISA to quantify the levels of IFN-α, IFNλ3, TNF-α, IL-6 and IL-1β (Figs. 4C–G). The results indicated that SVV infections led to a significant increase (*P* < 0.05 or *P* < 0.01) in the levels of IFN-α, IFNλ3, TNF-α, IL-6 and IL-1β compared to the control group. In contrast, adding Cu to the SVV-infected PK-15 cells significantly down-regulated (*P* < 0.05 or *P* < 0.01) cytokines. Conversely, the addition of TTM resulted in a significant increase (*P* < 0.05) of IL-6, IL-1β, IFN-α, and IFNλ3 levels, while TNF-α levels did not show a significant reduction (*P* > 0.05). These findings are consistent with the results presented in Figure 3C, suggesting that copper inhibits the NF-κB signalling pathways and cytokines activated by SVV, thereby promoting SVV replication.

### Mfn2 inhibits the NF-κB signalling pathway to promote SVV replication in PK-15 cells

**Figure 5:**
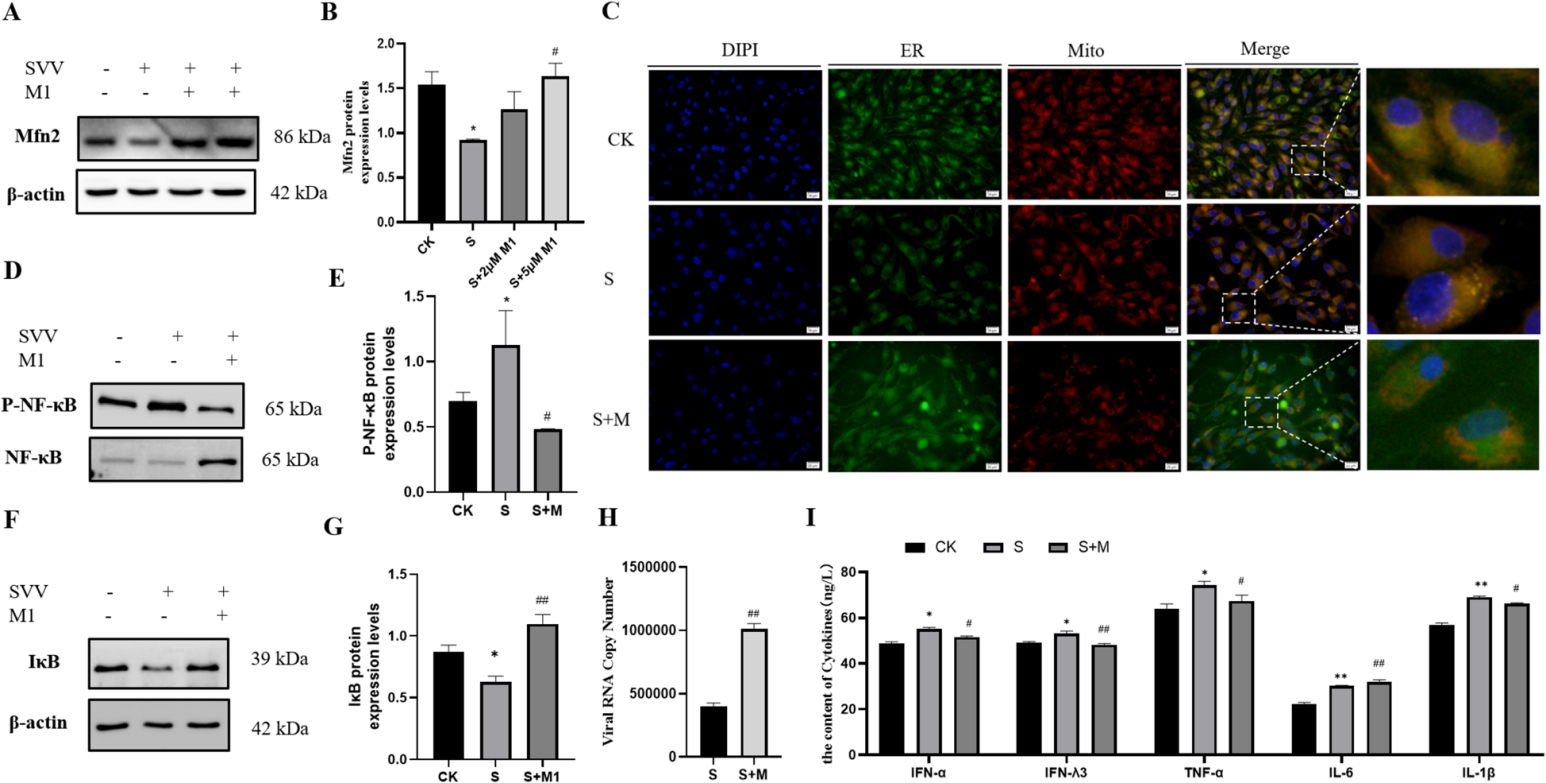
**Upregulated Mfn2 inhibits the NF-κB signalling pathway and promotes SVV replication in PK-15 cells. “M1” represents the mitochondrial fusion promoter M1. “S + M” stands for the SVV group with M1. A-B Protein expression levels of Mfn2 in PK-15 cells. C Immunoblot analysis of MAM in PK-15 cells. D-E Protein expression levels of p-NF-κB in PK-15 cells. F-G Protein expression levels of IκB in PK-15 cells. H Viral copy numbers of SVV in PK-15 cells (*n*** **= 9).** **I The levels of IL-1β, IL-6, TNF-α, IFN-α, and IFN-λ3 (*n*** = 3). Data are presented as means ± standard deviation. *  < 0.05, **P P **< 0.01 compared with the control group. #* P*** **< 0.05, ##* P*** **< 0.01 compared with the SVV group.**

Based on the results discussed above, we conclude that the steady-state imbalance of copper ions can affect SVV replication by modulating the MAM and NF-κB pathways. Next, we investigate the interaction between the MAM and NF-κB pathways during SVV infection in PK-15 cells. As shown in Figures 5A-C, Western blot analysis indicates that 5 μM M1 (mfn2 activator) increases (*P* < 0.05) Mfn2 protein levels and inhibits the connections between mitochondrial and ER membranes when compared to the SVV group. In contrast, 5 μM M1 significantly reduces the expression of p-NF-κB protein (Figures 5D and E). However, after SVV infects PK-15 cells, we observe a decrease in IκB protein levels, while the S + M group shows an increase in protein abundance (Figures 5F–G).

The ELISA assay indicated that in the S + M group, levels of IFN-α, IFNλ3, TNF-α, and IL-1β were significantly elevated (P < 0.05 or P < 0.01) compared to the SVV group. However, there was a notable decrease in IL-6 levels (*P* < 0.01). Additionally, we measured the viral copy number of SVV. We found that in PK-15 cells infected with SVV (Figure 5H), the addition of M1 substantially increased (*P* < 0.01) viral replication compared to the SVV group. Thus, the upregulation of Mfn2 can effectively inhibit the NF-κB signalling pathway and suppress cytokine production.

**Figure 6:**
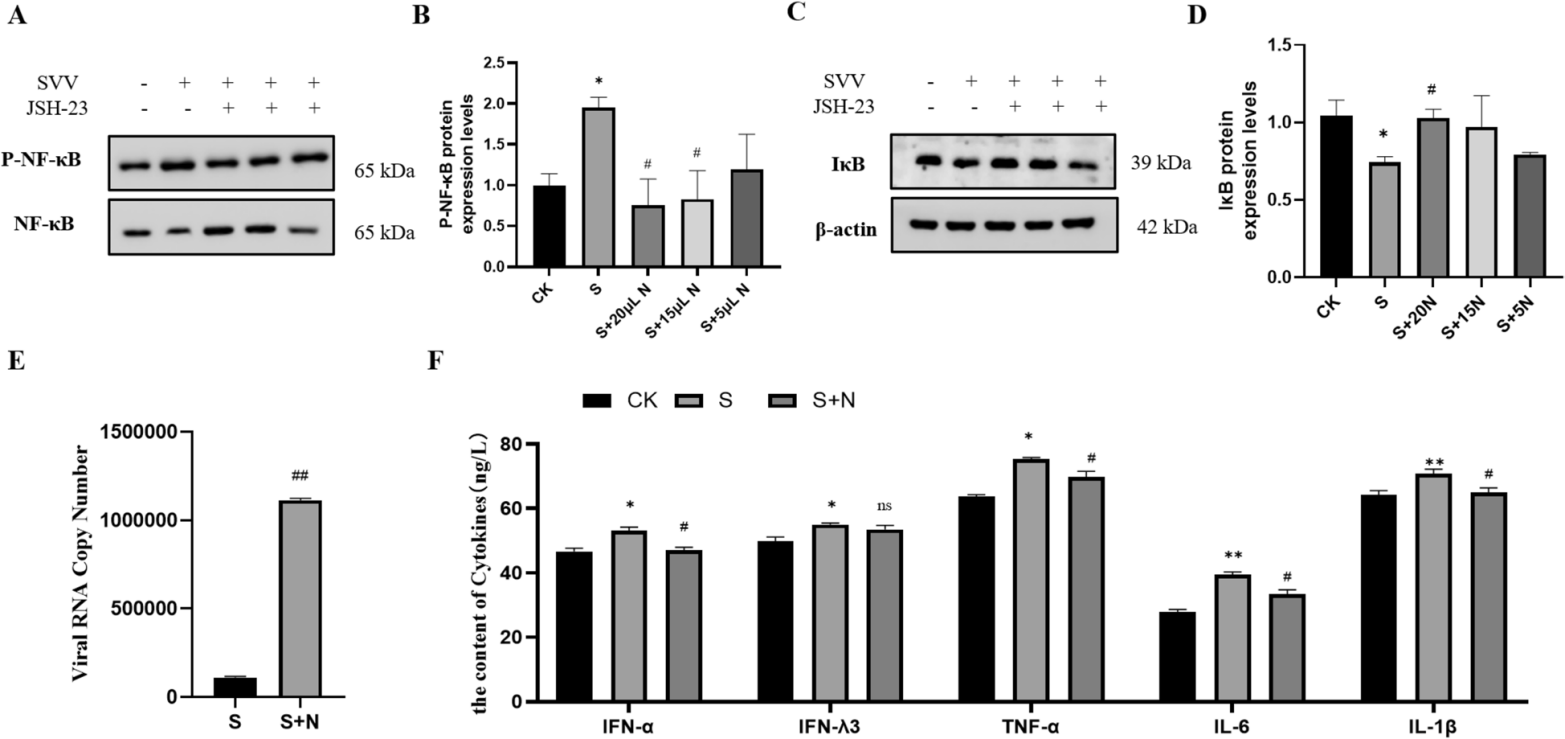
**Inhibiting the NF-κB signalling pathway affects SVV replication in PK-15 cells. “N” indicates JSH-23 treatment, which is an inhibitor of NF-κB “S + N” means the SVV group with N. A-B Protein expression levels of Mfn2 in PK-15 cells. C-D Protein expression levels of IκB in PK-15 cells. E Viral RNA copy numbers of SVV in PK-15 cells (*n*** **= 9). F The levels of IL-1β, IL-6, TNF-α, IFN-α, and IFN-λ3 (*n*** = 3). Data are presented as means ± standard deviation. * P < 0.05, ** P **< 0.01 compared with the control group. #* P*** **< 0.05, ##* P*** **< 0.01 compared with the SVV group.**

To further confirm the role of NF-κB in SVV replication, JSH23 (N), an inhibitor of the NF-κB signalling pathway, was administered to SVV-infected cells. Western blot analysis showed a significant reduction in p-NF-κB protein levels (*P* < 0.05) at 20 and 15 μM JSH23 (Figures 6A and B). Based on these findings, 20 μM JSH23 was selected for further experiments. Figure 6C demonstrates a marked increase in the copy number of SVV-infected cells upon the addition of the inhibitor (*P* < 0.01). In Figure 6D, with the exception of IFN-λ3, the levels of IL-1β, IL-6, TNF-α, and IFN-α in JSH23-treated PK-15 cells significantly decreased (*P* < 0.05 or *P* < 0.01) compared to the SVV group. These results indicate that inhibiting the NF-κB signalling pathway promotes virus replication in SVV-infected PK-15 cells.

## Discussion

SVV has emerged as a significant swine viral disease over the past two decades, presenting vesicular symptoms on the skin of pigs skin [26] Concurrently, numerous new viral lineages are continually evolving, exacerbating the global spread of SVV [27].

Recent research has attracted considerable attention to the relationship between copper and viral replication. Copper plays an integral role in many biological processes, and its regulation is complex, involving the interplay of various transport proteins (such as CTR1 and ATP7A/ATP7B), chaperones (including CCS, COX17, and ATOX1), and regulatory factors [28–30]. These components work together to ensure that copper is delivered to its necessary locations within the cell while also being sequestered from sensitive cellular compartments when not in use. Interestingly, certain viruses are susceptible to copper [31].

In light of this, we initially investigated how SVV affects the copper transport system in PK-15 cells. Our results showed that following SVV infection, the influx of copper ions through the key channel protein CTR1 into the cells increased, while the transport of copper ions facilitated by ATOX1 and ATP7B—which are responsible for transferring copper across the Golgi network and into the bloodstream—decreased. Cu is primarily stored in the mitochondria. The presence of SVV leads to a reduction in the protein levels of COX17, which in turn disrupts copper homeostasis, resulting in an increased concentration of copper ions in PK-15 cells. Chelating extra copper ions in these cells can partially inhibit SVV replication. This suggests that copper may promote SVV replication in PK-15 cells.

MAM undergo dynamic changes characterised by continuous division and fusion, and any imbalance in these processes has a significant impact on viral infections [32]. During the acute infection of hepatitis C virus (HCV), viral RNA is recognised by PRRs in host cells, interacting with MAVS within MAM, which aids in the body’s defence against HCV [33].

Several studies have demonstrated that knocking out the Mfn2 protein in SH-SY5Y cells significantly increases the number of MAMs [34, 35]. Conversely, other evidence suggests that suppressing Mfn2 increases the distance between the ER and mitochondria, damaging MAM integrity [36]. Despite this evidence, the role of Mfn2 in modulating the structure and function of MAM remains controversial.

In the present study, we showed that PK-15 cells infected with SVV exhibit reduced expression of the key regulatory protein Mfn2 in MAM. Additionally, SVV appears to enhance the connection between the ER and mitochondria. To explore whether Cu can regulate MAM and subsequently affect SVV replication, we treated SVV-infected PK-15 cells with 100 μM Cu^2+^ or 1 mM TTM. The results revealed that copper treatment increased Mfn2 protein expression and enhanced the distance between mitochondria and the ER in the SVV-infected group. Conversely, when we chelated excessive copper ions within the cells using TTM, the expression level of Mfn2 decreased, the distance between the mitochondria and the ER narrowed, and the interaction area expanded.

In conclusion, MAM in SVV-induced PK-15 cells is influenced by the imbalance of copper ion homeostasis, which affects SVV replication.

The NF-κB pathway is a vital component of cellular signal transduction and plays a pivotal role in regulating various physiological processes, including immune responses, inflammatory responses, and cell growth and death [37]. During viral infections, NF-κB, which is initially bound to IκB, is released. This pathway can respond to multiple stimuli; phosphorylated NF-κB forms homodimers that are transported to the nucleus, where they bind to IFN-stimulated response elements (ISREs) to induce the production of IFNs and inflammatory cytokines [38, 39], which in turn mounts an antiviral response.

Following NDV infection, the NF-κB pathway is activated, allowing p65 to enter the nucleus and mediate the release of cytokines such as IFN-β, IL-6, and IL-8, thereby enhancing the antiviral immune response and inhibiting viral replication [40]. In contrast, CoV-2 N cells infected with SARS inhibit the activation of the NF-κB pathway, which reduces the expression of interferons and inflammatory factors, thus promoting viral replication [41].

In our study, we found that infection of PK-15 Cells with SVV activated the NF-κB signalling pathway, characterised by the phosphorylation of NF-κB. This activation resulted in a decrease in the protein abundance of IκB and an increase in the expression of IL-1β, IL-6, TNF-α, IFN-α, and IFN-λ3.

We then inhibited the NF-κB signalling pathway using the inhibitor JSH-23. Following this inhibition, we observed a significant increase in the protein expression level of IκB and a rise in the copy number of SVV. In contrast, the levels of IL-1β, IL-6, TNF-α, and IFN-α were substantially lower compared to the SVV-infected group. These results suggest that NF-κB acts as a negative regulator of SVV replication.

Furthermore, Cu-binding peptides have been reported to exhibit anti-inflammatory activity in both BV-2 and primary microglia cultures by suppressing the NF-kB pathway [42]. Our experimental results from the experiment at 3.3 indicated that copper Cu enhances SVV replication. However, when Cu was added to PK-15 cells infected with SVV, we observed a decrease in the expression of p-NF-κB protein, along with lower levels of IFNs and inflammatory factors compared to the SVV group.

Conversely, when TTM was introduced, the outcomes reversed. These findings suggest that Cu can suppress the NF-κB signalling pathway activated by SVV, thereby reducing the production of IFNs and inflammatory factors, which allows the virus to evade the host immune response and facilitates SVV replication.

These results indicate that an imbalance in copper ion homeostasis can regulate viral replication by modulating both the MAM and NF-κB pathways. However, it is still unclear whether a specific connection exists between the MAM and the NF-κB pathway during SVV infection.

To investigate the mechanisms by which SVV regulates MAM and impacts the NF-κB pathway, we treated SVV-infected PK-15 cells with the Mfn2 activator M1. In this study, the addition of 5 μM M1 to SVV-infected PK-15 cells activated Mfn2 expression but reduced the interaction area between mitochondria and the ER compared to the SVV group without treatment.

Furthermore, the activation of Mfn2 stimulates the production of IκB, which binds to NF-κB and subsequently inhibits the SVV-activated NF-κB pathway. This inhibition results in a significant decrease in the levels of pro-inflammatory cytokines, including IL-1β, IL-6, TNF-α, and IFN-α, thereby diminishing the antiviral response.

These findings suggest that in SVV-infected PK-15 cells, a greater distance between the ER and mitochondria leads to a smaller interaction area, which exacerbates damage to MAMs. This, in turn, affects the NF-κB pathway, reducing the production of interferons and inflammatory factors, ultimately promoting SVV replication. Our research indicates that the disruption of copper ion homeostasis affects MAM, modulating the NF-κB signalling pathway and influencing viral replication in SVV-infected PK-15 cells. 

## Data Availability

The data underlying this article are available in the article and in its online supplementary material.

## References

[1] Zhang X, Zhu Z, Yang F, Cao W, Tian H, Zhang K, Zheng X, Liu H (2018) Review of Seneca Valley virus: a call for increased surveillance and research. Front Microbiol 9:940. 10.3389/fmicb.2018.00940 29867849 10.3389/fmicb.2018.00940 PMC5958643

[2] Zhang J, Zhang H, Sun W, Jiao C, Xiao P, Han J, Nan F, Xie C, Ha Z, Li Z, Xie Y, Meng Y, Lu H, Jin N (2021) Genetic evolution and epidemiological analysis of Seneca Valley virus (SVV) in China. Virus Res 291:198177. 10.1016/j.virusres.2020.198177 33038460 10.1016/j.virusres.2020.198177

[3] Xue Q, Liu H, Zhu Z, Yang F, Xue Q, Cai X, Liu XZheng H, (2018) Seneca Valley Virus 3C protease negatively regulates the type I interferon pathway by acting as a viral deubiquitinase. Antiviral Res 160:183–189. 10.1016/j.antiviral.2018.10.028 30408499 10.1016/j.antiviral.2018.10.028 PMC7111287

[4] Qian S, Fan W, Qian P, Chen H, Li X (2016) Isolation and full-genome sequencing of Seneca Valley virus in piglets from China, 2016. Virol J 13:173. 10.1186/s12985-016-0631-2 27756396 10.1186/s12985-016-0631-2 PMC5069920

[5] Prohaska JR (2008) Role of copper transporters in copper homeostasis. Am J Clin Nutr 88:826S-S829. 10.1093/ajcn/88.3.826S 18779302 10.1093/ajcn/88.3.826S PMC2799992

[6] Boyd SD, Ullrich MS, Skopp A, Winkler DD (2020) Copper sources for sod1 activation. Antioxidants (Basel) 9:500. 10.3390/antiox9060500 32517371 10.3390/antiox9060500 PMC7346115

[7] Mercer SW, Wang J, Burke R (2017) In vivo modeling of the pathogenic effect of copper transporter mutations that cause menkes and wilson diseases, motor neuropathy, and susceptibility to Alzheimer’s disease. J Biol Chem 292:4113–4122. 10.1074/jbc.M116.756163 28119449 10.1074/jbc.M116.756163 PMC5354492

[8] Zhu Z, Song M, Ren J, Liang L, Mao G, Chen M (2024) Copper homeostasis and cuproptosis in central nervous system diseases. Cell Death Dis 15:850. 10.1038/s41419-024-07206-3 39567497 10.1038/s41419-024-07206-3 PMC11579297

[9] Zhang S, Huang Q, Ji T, Li Q, Hu C (2024) Copper homeostasis and copper-induced cell death in tumor immunity: implications for therapeutic strategies in cancer immunotherapy. Biomark Res 12:130. 10.1186/s40364-024-00677-8 39482784 10.1186/s40364-024-00677-8 PMC11529036

[10] Kawahara M, Kato-Negishi M, Tanaka KI (2023) Dietary trace elements and the pathogenesis of neurodegenerative diseases. Nutrients 15:2067. 10.3390/nu15092067 37432185 10.3390/nu15092067 PMC10180548

[11] Yu CY, Liang JJ, Li JK, Lee YL, Chang BL, Su CI, Huang WJ, Lai MM, Lin YL (2015) Dengue virus impairs mitochondrial fusion by cleaving mitofusins. PLoS Pathog 11:e1005350. 10.1371/journal.ppat.1005350 26717518 10.1371/journal.ppat.1005350 PMC4696832

[12] Jaryal R, Khan SA (2023) Liquid-assisted mechanochemical synthesis, crystallographic, theoretical and molecular docking study on HIV instasome of novel copper complexes: (µ-acetato)-bis(2,2’-bipyridine)-copper and bromidotetrakis(2-methyl-1H-imidazole)-copper bromide. Biometals 36:975–996. 10.1007/s10534-023-00498-6 37010713 10.1007/s10534-023-00498-6

[13] Rupp JC, Locatelli M, Grieser A, Ramos A, Campbell PJ, Yi H, Steel J, Burkhead JL, Bortz E (2017) Host cell copper transporters CTR1 and ATP7A are important for influenza A virus replication. Virol J 14:11. 10.1186/s12985-016-0671-7 28115001 10.1186/s12985-016-0671-7 PMC5259989

[14] Puig-Pijuan T, Souza LRQ, Cdsg P, Higa LM, Monteiro FL, Tanuri A, Valverde RHF, Einicker-Lamas M, Rehen SK (2022) Copper regulation disturbance linked to oxidative stress and cell death during Zika virus infection in human astrocytes. J Cell Biochem 123:1997–2008. 10.1002/jcb.30323 36063501 10.1002/jcb.30323

[15] Ishii KJ, Koyama S, Nakagawa A, Coban C, Akira S (2008) Host innate immune receptors and beyond: making sense of microbial infections. Cell Host Microbe 3:352–363. 10.1016/j.chom.2008.05.003 18541212 10.1016/j.chom.2008.05.003

[16] Downton P, Bagnall JS, England H, Spiller DG, Humphreys NE, Jackson DA, Paszek P, White MRH, Adamson AD (2023) Overexpression of IκB⍺ modulates NF-κB activation of inflammatory target gene expression. Front Mol Biosci 10:1187187. 10.3389/fmolb.2023.1187187 37228587 10.3389/fmolb.2023.1187187 PMC10203502

[17] Liu T, Li X, Wu M, Qin L, Chen H, Qian P (2019) Seneca valley virus 2C and 3C pro induce apoptosis via mitochondrion-mediated intrinsic pathway. Front Microbiol 10:1202. 10.3389/fmicb.2019.01202 31191506 10.3389/fmicb.2019.01202 PMC6549803

[18] Horner SM, Liu HM, Park HS, Briley Jgale M (2011) Mitochondrial-associated endoplasmic reticulum membranes (MAM) form innate immune synapses and are targeted by hepatitis C virus. Proc Natl Acad Sci U S A 108:14590–14595. 10.1073/pnas.1110133108 21844353 10.1073/pnas.1110133108 PMC3167523

[19] Li J, Qi F, Su H, Zhang C, Zhang Q, Chen Y, Chen P, Su L, Chen Y, Yang Y, Chen Z, Zhang S (2022) GRP75-faciliated mitochondria-associated ER membrane (MAM) integrity controls cisplatin-resistance in ovarian cancer patients. Int J Biol Sci 18:2914–2931. 10.7150/ijbs.71571 35541901 10.7150/ijbs.71571 PMC9066115

[20] Eura Y, Ishihara N, Yokota S, Mihara K (2003) Two mitofusin proteins, mammalian homologues of FZO, with distinct functions are both required for mitochondrial fusion. J Biochem 134:333–344. 10.1093/jb/mvg150 14561718 10.1093/jb/mvg150

[21] Filadi R, Pendin D, Pizzo P (2018) Mitofusin 2: from functions to disease. Cell Death Dis 9:330. 10.1038/s41419-017-0023-6 29491355 10.1038/s41419-017-0023-6 PMC5832425

[22] Kim IS, Silwal P, Jo EK (2021) Mitofusin 2, a key coordinator between mitochondrial dynamics and innate immunity. Virulence 12:2273–2284. 10.1080/21505594.2021.1965829 34482801 10.1080/21505594.2021.1965829 PMC8425681

[23] Gou H, Zhao M, Xu H, Yuan J, He W, Zhu M, Ding H, Yi L, Chen J (2017) CSFV induced mitochondrial fission and mitophagy to inhibit apoptosis. Oncotarget 8:39382–39400. 10.18632/oncotarget.17030 28455958 10.18632/oncotarget.17030 PMC5503620

[24] Ichinohe T, Yamazaki T, Koshiba T, Yanagi Y (2013) Mitochondrial protein mitofusin 2 is required for NLRP3 inflammasome activation after RNA virus infection. Proc Natl Acad Sci U S A 110:17963–17968. 10.1073/pnas.1312571110 24127597 10.1073/pnas.1312571110 PMC3816452

[25] Deng H, Zhu S, Zhu L, Sun J, Ding Y, Li F, Jian Z, Zhao J, Deng L, Deng J, Deng Y, Guo H, Sun X, Lai SY, Tang H, Cui H, Ge LPXuZ (2022) Mfn2 is responsible for inhibition of the RIG-I/IRF7 pathway and activation of NLRP3 inflammasome in Seneca Valley virus-infected PK-15 cells to promote viral replication. Front Immunol 13:955671. 10.3389/fimmu.2022.955671 35958608 10.3389/fimmu.2022.955671 PMC9359100

[26] He W, Zhao J, Xing G, Li G, Wang R, Wang Z, Zhang C, Franzo G, Su S, Zhou J (2019) Genetic analysis and evolutionary changes of Porcine circovirus 2. Mol Phylogenet Evol 139:106520. 10.1016/j.ympev.2019.106520 31152778 10.1016/j.ympev.2019.106520

[27] Buckley AC, Michael DD, Faaberg KS, Guo B, Yoon KJ, Lager KM (2021) Comparison of historical and contemporary isolates of Senecavirus A. Vet Microbiol 253:108946. 10.1016/j.vetmic.2020.108946 33341466 10.1016/j.vetmic.2020.108946

[28] Saeng-Chuto K, Rodtian P, Temeeyasen G, Wegner M, Nilubol D (2018) The first detection of Senecavirus A in pigs in Thailand, 2016. Transbound Emerg Dis 65:285–288. 10.1111/tbed.12654 28474854 10.1111/tbed.12654

[29] Arzt J, Bertram MR, Vu LT, Pauszek SJ, Hartwig EJ, Smoliga GR, Palinski R, Stenfeldt C, Fish IH, Hoang BH, Phuong NT, Hung VV, Vu PP, Dung NK, Dong PV, Tien NN, Dung DH (2019) First detection and genome sequence of senecavirus A in Vietnam. Microbiol Resour Announc 8:e01247–18. 10.1128/mra.01247-18 30687818 10.1128/MRA.01247-18 PMC6346150

[30] Liu J, Ren X, Li Z, Xu G, Lu R, Zhang K, Ning Z (2018) Genetic and phylogenetic analysis of reemerged novel Seneca Valley virus strains in Guangdong province, 2017. Transbound Emerg Dis 65:614–617. 10.1111/tbed.12839 29461010 10.1111/tbed.12839

[31] Borkow G, Lara HH, Covington CY, Nyamath A, Gabbay J (2008) Deactivation of human immunodeficiency virus type 1 in medium by copper oxide-containing filters. Antimicrob Agents Chemother 52:518–525. 10.1128/aac.00899-07 18070974 10.1128/AAC.00899-07 PMC2224774

[32] Rieusset J (2018) Mitochondria-associated membranes (MAMs): An emerging platform connecting energy and immune sensing to metabolic flexibility. Biochem Biophys Res Commun 500:35–44. 10.1016/j.bbrc.2017.06.097 28647358 10.1016/j.bbrc.2017.06.097

[33] Sumpter R, Loo YM, Foy E, Li K, Yoneyama M, Fujita T, Lemon SM, Gale M (2005) Regulating intracellular antiviral defense and permissiveness to hepatitis C virus RNA replication through a cellular RNA helicase, RIG-I. J Virol 79:2689–2699. 10.1128/jvi.79.5.2689-2699.2005 15708988 10.1128/JVI.79.5.2689-2699.2005 PMC548482

[34] de Brito OM, Scorrano L (2008) Mitofusin 2 tethers endoplasmic reticulum to mitochondria. Nature 456:605–610. 10.1038/nature07534 19052620 10.1038/nature07534

[35] Sebastián D, Hernández-Alvarez MI, Segalés J, Sorianello E, Muñoz JP, Sala D, Waget A, Liesa M, Paz JC, Gopalacharyulu P, Orešič M, Pich S, Burcelih R, Palacín M, Zorzano A (2012) Mitofusin 2 (Mfn2) links mitochondrial and endoplasmic reticulum function with insulin signaling and is essential for normal glucose homeostasis. Proc Natl Acad Sci U S A 109:5523–5528. 10.1073/pnas.1108220109 22427360 10.1073/pnas.1108220109 PMC3325712

[36] Filadi R, Greotti E, Turacchio G, Luini A, Pozzan T, Pizzo P (2015) Mitofusin 2 ablation increases endoplasmic reticulum-mitochondria coupling. Proc Natl Acad Sci U S A 112:E2174–E2181. 10.1073/pnas.1504880112 25870285 10.1073/pnas.1504880112 PMC4418914

[37] Taniguchi Kkarin M (2018) NF-κB, inflammation, immunity and cancer: coming of age. Nat Rev Immunol 18:309–324. 10.1038/nri.2017.142 29379212 10.1038/nri.2017.142

[38] Zheng J, Shi W, Yang Z, Chen J, Qi A, Yang Y, Deng Y, Yang D, Song N, Song B, Luo D (2023) RIG-I-like receptors: molecular mechanism of activation and signaling. Adv Immunol 158:1–74. 10.1016/bs.ai.2023.03.001 37453753 10.1016/bs.ai.2023.03.001

[39] Sadler AJ, Williams BR (2008) Interferon-inducible antiviral effectors. Nat Rev Immunol 8:559–568. 10.1038/nri2314 18575461 10.1038/nri2314 PMC2522268

[40] Wang H, Feng G, Liao Y (2017) NDV activates NF-κB pathway through PKR to induce the expression of IFN-β and inflammatory factor. J Nanjing Agricult Univ 40:488–493. 10.7685/jnau.201611038

[41] Guo X, Yang S, Cai Z, Zhu S, Wang H, Feng J, Chen X, Li Y, Deng J, Liu J, Li J, Tan X, Fu Z, Xu K, Zhou L, Chen Y (2025) SARS-CoV-2 specific adaptations in N protein inhibit NF-κB activation and alter pathogenesis. J Cell Biol 224:e202404131. 10.1083/jcb.202404131 39680116 10.1083/jcb.202404131 PMC11648720

[42] Caetano-Silva ME, Rund LA, Vailati-Riboni M, Pacheco Bertoldo MT, Johnson RW (2021) Copper-binding peptides attenuate microglia inflammation through suppression of NF-kB pathway. Mol Nutr Food Res 65:e2100153. 10.1002/mnfr.202100153 34532985 10.1002/mnfr.202100153 PMC8612997

